# Quantum Conductance in Silicon Oxide Resistive Memory Devices

**DOI:** 10.1038/srep02708

**Published:** 2013-09-19

**Authors:** A. Mehonic, A. Vrajitoarea, S. Cueff, S. Hudziak, H. Howe, C. Labbé, R. Rizk, M. Pepper, A. J. Kenyon

**Affiliations:** 1 Department of Electronic & Electrical Engineering, UCL, Torrington Place, London WC1E 7JE, UK; 2 Centre de Recherche sur les Ions, les Matériaux et la Photonique (CIMAP), UMR 6252 CNRS/CEA/Ensicaen/UCBN, 6 Boulevard Maréchal Juin, 14050 Caen Cedex 4, France; 3 Brown University, School of Engineering, Providence, Rhode Island 02912, USA

## Abstract

Resistive switching offers a promising route to universal electronic memory, potentially replacing current technologies that are approaching their fundamental limits. In many cases switching originates from the reversible formation and dissolution of nanometre-scale conductive filaments, which constrain the motion of electrons, leading to the quantisation of device conductance into multiples of the fundamental unit of conductance, G_0_. Such quantum effects appear when the constriction diameter approaches the Fermi wavelength of the electron in the medium – typically several nanometres. Here we find that the conductance of silicon-rich silica (SiO_x_) resistive switches is quantised in half-integer multiples of G_0_. In contrast to other resistive switching systems this quantisation is intrinsic to SiO_x_, and is not due to drift of metallic ions. Half-integer quantisation is explained in terms of the filament structure and formation mechanism, which allows us to distinguish between systems that exhibit integer and half-integer quantisation.

Devices in which the electrical resistance can stably and repeatedly be switched between vastly different values hold great potential as future non-volatile memory elements. So-called Resistive Random Access Memories (RRAMs) exploiting such switching behaviour are actively being developed as alternatives to current technologies such as Flash, which are approaching their limits of scalability and power dissipation^1^. We recently demonstrated switching in silicon oxide^2^,^3^, a material of great technological importance, silicon being the semiconductor of choice for microelectronics. Switching occurs in silicon oxide, as in many other resistive switching systems, by the reversible formation and dissolution of nanometre-scale conductive filaments^4^,^5^,^6^. These are constrictions through which electrons must flow under an externally applied field. At such small length scales quantum effects begin to dominate, and in the limit of the constriction diameter being of the order of the Fermi wavelength of the electron in the medium (typically nanometres), electron transport is quantised in multiples of the fundamental quantum of conductance G_0_ ( = 2*e*^2^/*h*, where *e* is the charge of the electron and *h* is Planck's constant). Under these conditions, the mean free path of the electron is greater than the diameter of the constriction, and electrons move through the constriction without scattering – ie *ballistically*. Such quantisation of conductance is a well-studied phenomenon, having been reported in a range of materials and systems^7^,^8^,^9^, though there are only a handful of reports relating to RRAM devices (see, for example, Zhu *et al.*^10^). Nevertheless, quantum conductance in such systems is of great interest not only because of the opportunity it affords to study the physics of electron transport and resistive switching, but also because it has potential applications in multilevel semiconductor memories, quantum information processing, and neuromorphic systems.

Reports in the literature of quantum conductance in RRAM devices variously mention quantisation in integer or half-integer multiples of G_0_. There is a need to reconcile these results, to understand which devices exhibit half-integer quantisation, and to relate this to the physics of the resistance switching process. Here we report quantum conductance in silicon oxide-based RRAM devices in which multilevel conductance steps are separated by half-integer multiples of G_0_. Conductance steps are likely to be associated with individual conductive filaments, as is the case in metal oxide systems. However, the more complex carrier transport in our devices, along with the inherent nonlinearity of our semiconductor system, allows us to determine the origin of the half-integer quantisation in these and other RRAM devices.

## Results

### Resistive switching and conductance quantisation

**Figure 1:**
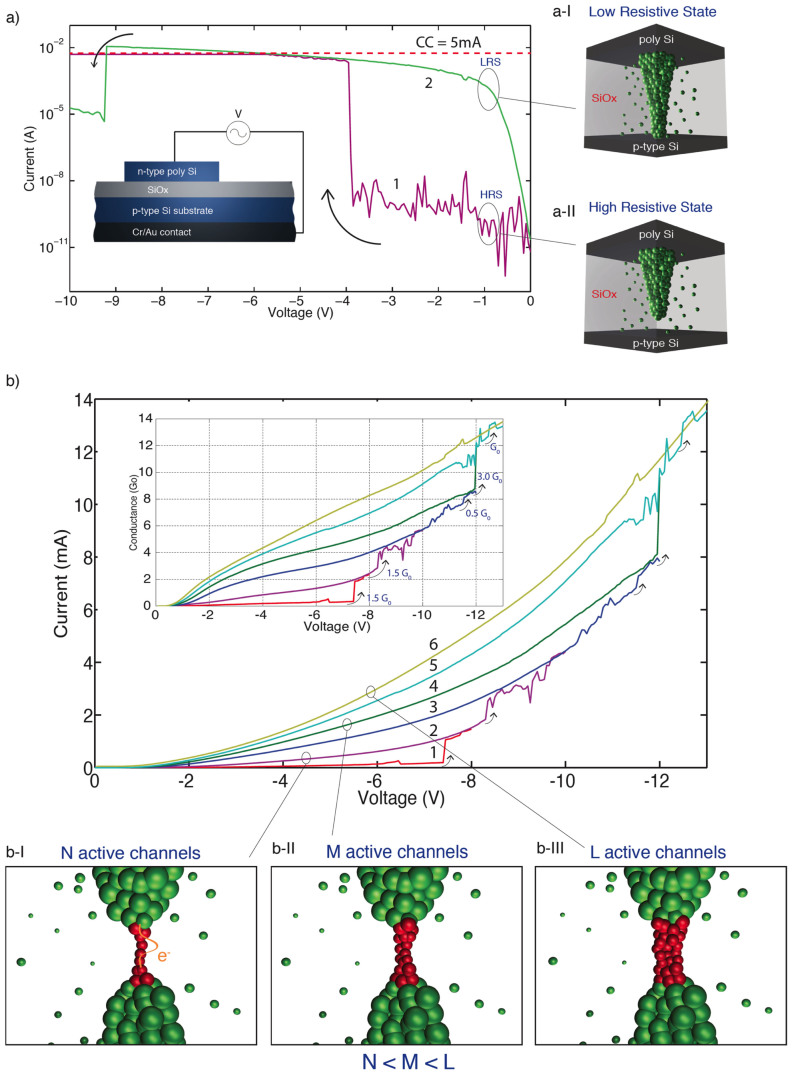
Unipolar switching of a device. (a) Magenta line: initial voltage sweep, with a current compliance (CC) limit of 5 mA, showing a transition from OFF to ON state at −4 V. Green line: subsequent voltage sweep of the ON state, with current compliance removed. Arrows indicate the direction of voltage sweep. Inset: Device schematic cross-section. (a-I) Schematic showing the formation of a continuous conductive filament within the oxide matrix, corresponding to the low resistive state of the device. (a-II) Schematic showing a break in the filament, corresponding to the high resistive state of the device. (b) Current-voltage characteristics of multiple conduction states during sequential voltage sweeps of a single device. Inset: corresponding conductance-voltage graph. (b-I, b-II, b-III) show schematics of the growth in filament thickness during successive voltage sweeps to progressively higher voltages. N, M and L are indices that denote the number of active channels in the constriction, with N < M < L.

Our devices, shown schematically in the inset of figure 1, consist of a thin (16 nm) silicon-rich silica (SiO_x_) layer sandwiched between a p-type silicon substrate and an n-type polycrystalline silicon top contact. They exhibit both bipolar and unipolar resistive switching, as discussed in our previous work^2^,^3^; here we concentrate on unipolar operation in which transitions between resistive states all occur for the same polarity of applied voltage. After an electroforming step (details in supplementary information), an initial High Resistive State (HRS) switches to a Low Resistive State (LRS) at a threshold voltage typically around −4 V (Figure 1a) by the reversible formation of a conductive filament within the insulating film^2^,^4^,^5^,^6^. In contrast to our previous work, in this study we set a current compliance limit to prevent hard breakdown of our devices. Our previous results^2^ showed that a number of devices exhibited anomalous unipolar switching behaviour in which V_reset_ was larger than V_set_. By setting a current compliance in this case we were able to obtain unipolar switching from a larger number of devices in which V_reset_ < V_set_, as is common in systems such as metal oxides. This enabled more stable unipolar operation than in the case when no current compliance is set. Our previous STM measurements of the diameter of conductive filaments at the top of the oxide layer show them to be in the range of a few nanometres to tens of nanometres^2^ – well within the range for which quantum effects should be observable. The filaments are formed by the drift of oxygen vacancies under the application of an external electric field between top and bottom electrodes, resulting in a single continuous or semi-continuous conducting filament bridging the two electrodes^11^. Filamentary resistive switching occurs at a weak (narrow) point. Such a point presents a quantum constriction, leading to one-dimensional confinement of carriers during transport, and consequent quantisation of conductance. Importantly, devices can repeatedly be switched back and forth between HRS and LRS by the application of suitable voltages^2^.

**Figure 2:**
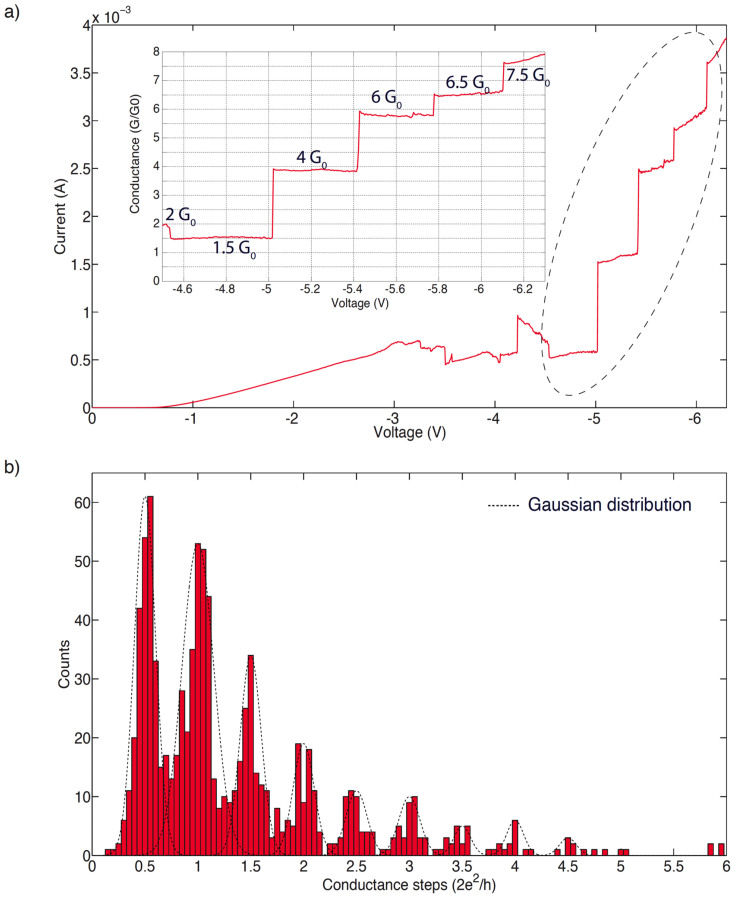
Quantised conductance steps. (a) Current-voltage and (inset) conductance-voltage curves for a device showing linear behaviour in the voltage range between −4.5 V and −6.1 V (expanded in the inset). Several level conductance plateaux can be seen at half-integer multiples of G_0_. Note the deviation from linearity in the G–V curve above 6.1 V and G = 7.5 G_0_. (b) Histogram of conductance changes during ~1,000 conductance steps. Clear peaks are evident at half-integer multiples of G_0_, which have been fitted with a series of Gaussian distributions as a guide to the eye (dotted lines).

After switching our device to a LRS we applied a sequence of voltage sweeps to progressively higher voltages, during each of which multiple current jumps were seen, as shown in Figure 1(b). These jumps are much smaller than the transition between the HRS and LRS, which can be around five orders of magnitude. Note that the device reset current depends on the current compliance applied during the initial set process (as described in reference 16). In the case of the results shown in Figure 1(a) a current compliance of 5 mA was used, while for those reported in Figure 1(b) no current compliance was set. This explains why reset occurs at 10 mA in the former case (reset current scales with current compliance, but the two are not necessarily equal), but is not seen even for higher currents than this in the latter. We found also that the overall conductance slightly increased with each consecutive sweep. This may be due to a progressive thickening of the conductive filament, as illustrated schematically in Fig. 1(b). As the voltage is swept to progressively higher and higher values, more and more oxygen ions are removed from the region surrounding the filament, uncovering a larger volume of conductive tissue^3^. We gradually increased the maximum voltage bias until no further abrupt changes were detected, corresponding to a fully formed conductive path with no further filament thickening. Note that it is possible to return devices to the high resistive state following such a sequence of voltage sweeps, and devices can be cycled many times between high resistive and low resistive states, exhibiting stable unipolar operation after many set and reset cycles. Nevertheless, current compliance level set during switching greatly influences device endurance; both endurance and stability of switching is improved by setting an appropriate current compliance (3–5 mA) during the set process. During these scans around one thousand abrupt conduction steps were recorded, and the distribution of their conductances plotted in figure 2(b). The histogram can be fitted with a set of normal distributions with peaks positioned at half-integer multiples of the quantum conductance unit G_0_, confirming the signature of ballistic carrier transport in a one-dimensional constriction.

### Nonlinear components of the conductance

The nonlinear conductance curves in figure 1(b) suggest contributions from both linear and nonlinear conduction channels, the former of which exhibits quantisation (more details are given in Supplementary Information). The degree of linearity of device response depends strongly on the strength of the conductive filament – those supporting higher currents tend to be more linear, in keeping with our previous observations^3^. In the case of unipolar switching of SiO_x_, when sufficient current flows through the filaments their behaviour becomes more Ohmic, suggesting the formation of a highly conductive phase of silicon, or of a mini-band in the silicon oxide band gap associated with dangling bonds or oxygen vacancies; devices become progressively more linear as the voltage is swept to ever higher voltages. The observation of a conductive phase of silicon is supported by TEM data from Yao *et al*.^12^, who suggest on the basis of high-resolution Transmission Electron Microscopy studies that local Joule heating can transform the silicon filament into a highly conductive Si-II/Si-XII phase that has a much lower resistivity than that of bulk silicon. The formation of a dangling bond related mini band is supported by results from Wang *et al.*^13^. Whichever is the case, it is clear that the Ohmic contribution to conductance comes from a conductive material phase whose linearity depends on the past history of applied voltages. We assume that the material within the constriction is the same as the material forming the bulk of the conductive filament.

We show in figure 2(a) current–voltage (I–V) and conductance–voltage (G–V) results from a highly linear device. In this case, beyond an initial threshold at around 0.75 V, which may be due to a potential barrier at one of the interfaces between the filament and the electrodes, the flat–topped steps in the G–V curve that indicate linearity occur clearly at half-integer multiples of G_0_ for applied voltages between −4.5 V and −6.1 V. Above this the curve becomes nonlinear.

Our results imply that device conductance may be expressed as a sum of linear and nonlinear terms: 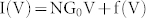
where f(V) represents a nonlinear background current through semiconducting tissue. This background conduction does not significantly change during successive voltage sweeps. Instead, we see a set of discrete, quantised jumps in the linear component. We may suppose that the Ohmic contribution to conductance comes from a highly conductive filament core, which may be either the Si-II/Si-XII silicon phase suggested by Yao *et al*., or else a mini-band within the oxide matrix, while the nonlinear component may arise from an interface region around the filament within which phase separation of the conductive channel has not fully progressed. Further details are given in Supplementary Information.

## Discussion

**Figure 3:**
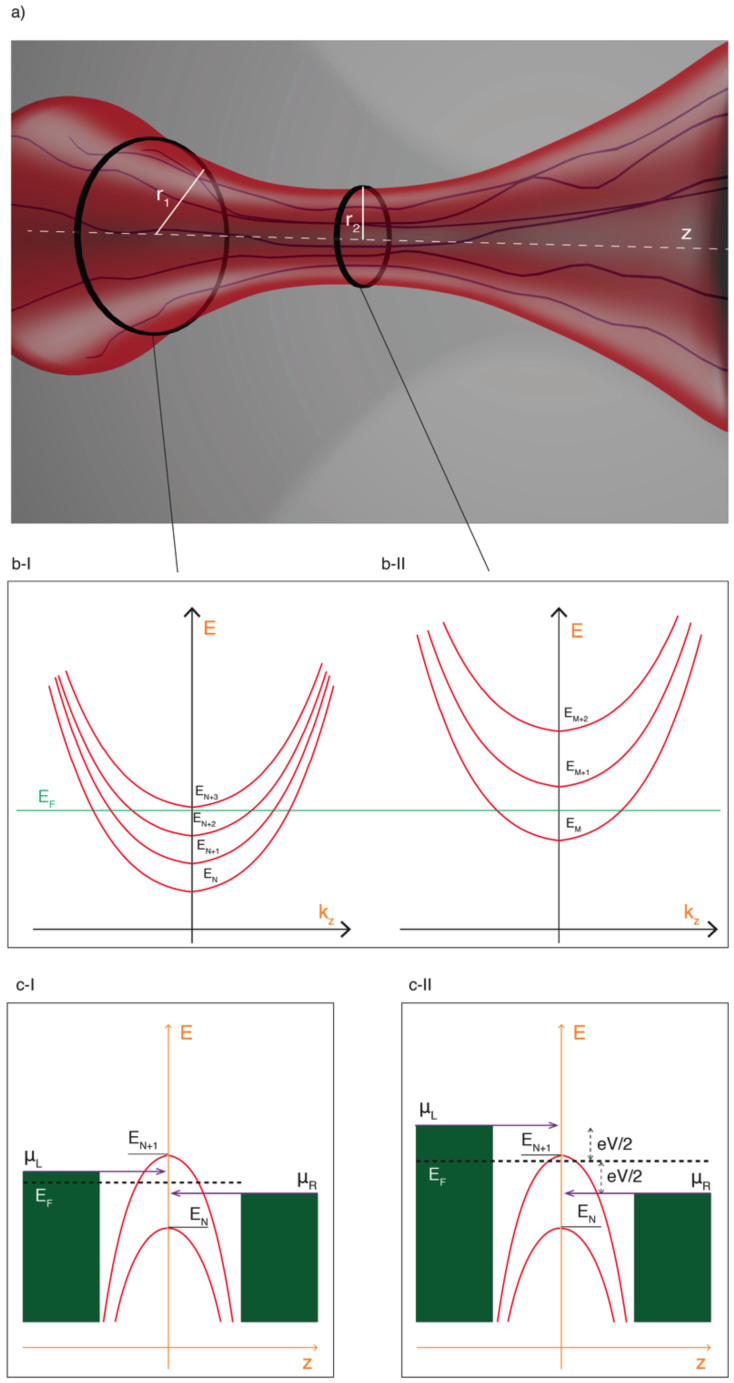
The effect of a quantum constriction on conductance. (a) schematic diagram of the quantum constriction at the heart of the filament. (b-I) illustrates the dispersion curve of the first four electronic sub-bands at the edge of the constriction. (b-II) shows the first three sub-bands at the centre of the constriction where the confinement is stronger, causing a spacing-out of the sub-bands. (c-I) shows the situation in which the difference in chemical potential between the L and R reservoirs is small, and hence the L- and R-going electron modes both fall within the same sub-band. In (c-II) there is a larger difference in chemical potentials (ie a higher bias), causing the L- and R-going modes to fall into different sub-bands.

The switching behaviour depicted in the I–V characteristics of the low resistance state (LRS), can be explained within the framework of Landauer theory for mesoscopic systems. The very high degree of lateral confinement of carriers within the quantum constriction (Figure 3(a)) produces a set of discrete one-dimensional sub-bands in the conduction band of the constriction, through which electrons flow^14^,^15^. This is illustrated schematically in Figure 3(b). When the spacing between the sub-bands is greater than k_B_T and the applied bias is greater than the sub-band energy spacing (and therefore also greater than k_B_T), steps are seen in the resulting conductance/voltage characteristics. As the width of the constriction increases, more conduction modes are allowed; for each additional mode the conductance of the device jumps by one unit of G_0_.

To account for the observed half-integer G_0_ plateaux we modify the preceding argument by considering the nonlinear quantum-point conduction model. We first assume that voltage drops equally at both sides of the quantum constriction shown in Figure 3(a). Importantly, a prerequisite for the observation of quantum conductance is for electron transport through the constriction to be ballistic, in which case no voltage is dropped within the constriction. Instead, an applied field generates a difference in chemical potentials between the left and right electron reservoirs. Following the finite-bias Landauer approach, the influx of electrons from each electrode is expressed in terms of the transmission probability of the constriction, the number of available conduction modes (sub-bands) and the Fermi distribution. The current coming from the left electrode (reservoir) is: 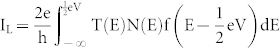
where T is the transmission probability, N(E) is the number of sub-bands in the constriction, f(E) is the Fermi distribution and V is the bias voltage. Similarly, the current coming from the right electrode is: 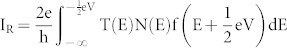


We approximate the transmission probability for electrons above the energy minimum of the sub-band to one, and that of the electrons with energy below this to zero. Adopting the zero temperature limit, currents from both sides are: 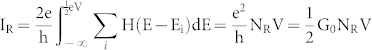

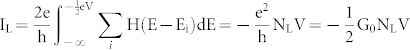
where N_R_ and N_L_ are the numbers of occupied sub-bands accessed from the right and left sides, respectively, and H(E) is the Heaviside unit step function. The total current is: 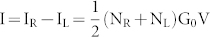


If N_R_ + N_L_ is an odd number quantised plateaux at half integer multiples of G_0_ will be observed rather than the usual integer values. In general, therefore, we can say that systems in which the difference in chemical potentials between the two electron reservoirs is small only integer quantisation of conductance will be seen.

In the case of our device, the voltage bias across the device prior to the observation of conductance steps is much higher than that reported in metal oxide based RRAM devices^16^. In this case the chemical potential of the left reservoir (μ_L_) can rise above the energy E_N+1_ while that of the right reservoir (μ_R_) will still be between E_N_ and E_N+1_, where E_N_ and E_N+1_ are minimum energies for the N^th^ and (N + 1)^th^ sub-bands. This is shown in figure 3(c-II). When there is an odd number of conduction modes transporting current in only one direction, half-integer G_0_ plateaux are observed.

Miranda *et al.*^14^ have provided a similar theoretical treatment for the electron transport in CeO_x_-based resistive switching devices. Early reports of the nonlinear quantum-point transport mode can be found in work of Glazman and Khaetskii^17^, Xu *et al.*^18^, and Patel *et al.*^7^.

Importantly, this model allows us to reconcile the various reports of integer- and half-integer quantisation of conductance observed in RRAM devices in the literature. Both are reported, with half-integer quantisation being variously ascribed to non-unity transmission probabilities, absorbed and adsorbed defects on metallic filaments, and scattering. There is thus some uncertainty surrounding the origin of half-integer quantisation – an uncertainty that needs to be resolved. It is clear from the preceding discussion that the key quantity governing the type of quantisation seen is the difference in chemical potentials between the two reservoirs (Δμ).

At this point we can consider in more detail the mechanism by which conductive filaments are formed during resistive switching. Broadly, there are two classes of resistive switching devices in which quantisation of conductance is reported: electrochemical metallisation (ECM) and valence change memory (VCM)^19^. In the former, conductive filaments form by the drift into the switching matrix of metal ions from one or other of the metallic electrodes. In the latter, application of an external field to the switching layer induces a redox reaction, resulting in the migration of oxygen vacancies within the active matrix, revealing a filament of conductive tissue connecting the two electrodes. In a sense, we may thus describe ECM switching as *extrinsic* to the switching medium, while VCM is *intrinsic*. In our devices, unipolar switching can be assigned to a third class of mechanism: thermochemical (TCM)^19^. In this case, although the initial HRS-LRS transition is driven by a redox reaction similar to that of VCM, the reset process is one of Joule heating – hence thermochemical. Nevertheless, we may still classify the switching as *intrinsic*.

**Figure 4:**
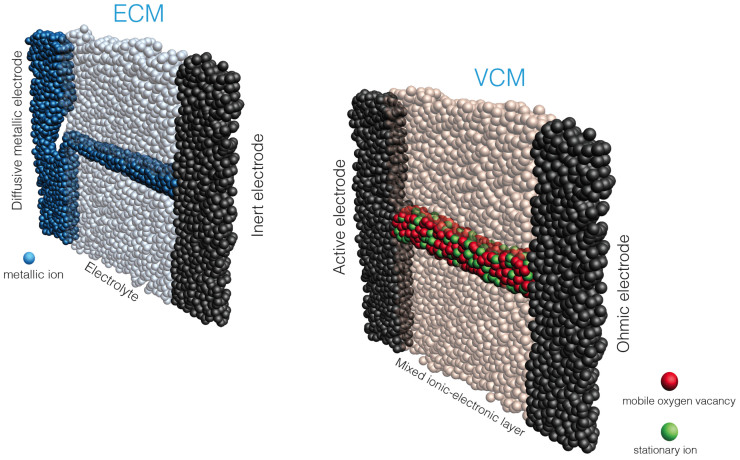
Schematic representation of conductive filaments formed in ECM (electrochemical metallisation) and VCM (valence change memory) systems. LHS: in electrochemical memory systems the conductive filament is a continuous metallic pathway formed by the drift of metal ions from an active electrode (for example, Ag) into the dielectric layer. RHS: in the case of valence change memories, drift of oxygen vacancies and associated redox reactions within the dielectric layer result in a more mixed filament, typically with a higher resistivity than that of ECM systems.

We now return to the issue of integer and half-integer quantisation of conductance – specifically, how this relates to the filament switching mechanism. Here we note the metallic nature of the filament in ECM devices. In the majority of reports of conductance quantisation in RRAM devices the filament is silver, and in all of these cases integer quantisation is seen. This is consistent with the impossibility of maintaining a large difference in chemical potential between the electron reservoirs in the case of a highly conductive filament. On the other hand, most VCM systems exhibit half-integer quantisation, implying that filaments formed by valence change mechanisms can sustain a large Δμ between the reservoirs. This is to be expected if we consider the formation of VCM filaments to be more stochastic than that of those resulting from ECM. The latter are driven by the sequential aggregation of metallic cations around an initial nucleation site on one electrode. Field-driven drift of metal ions results in a continuous metal filament bridging the electrodes. However, in VCM devices the growth of filaments is more uneven, and the resulting conductive pathway, in the case of metal oxides, is an admixture of metal cations and oxygen vacancies^19^. This is reflected in the higher resistivity reported for VCM devices – they do not generally require the setting of current limits during switching, in contrast to ECM devices that sustain much higher currents and hence are prone to breakdown if the current is not restricted. Figure 4 is a schematic representation of conductive filaments in ECM and VCM switching, emphasising the intermixing of cations and oxygen vacancies in the latter.

Table 1 summarises the literature reports of conductance quantisation in resistive switching devices. We note that the early reports of switching in amorphous silicon and in vanadium pentoxide^20^,^22^ did not specify switching mechanism, and the determination of filament type was unclear in both cases. Nevertheless, we can surmise that V_2_O_5_ is a VCM material, in line with other transition metal oxides, which is consistent with the observed half-integer quantisation of conductance. In the case of a-Si:H, although there is undoubtedly some contribution from metallic drift from the electrodes, it is likely that this is not the full story, given the complexity of the a-Si:H system. We can reasonably suppose that a more mixed filament is responsible for half-integer quantisation in this case as well, as indeed reported by the authors. In the case of hafnium oxide, the authors show linear conduction with high currents, which they ascribe to the formation of thick metallic filaments. This, again, is consistent with the observed integer quantisation of conductance.

Studying the quantisation of conductance in filamentary resistive switches offers an insight into the ultimate scaling limits of RRAM, as quantisation can only be seen when the diameter of the narrowest point of the filament (the quantum constriction) is smaller than the Fermi wavelength of the electron in the medium. Assuming the conductive filament in our devices to be silicon, the upper limit on the filament diameter is therefore between 35 nm and 112 nm^20^. This is consistent with our previous STM results that demonstrated the nanometre-scale size of conductive regions in a switched SiO_x_ film^2^.

In conclusion, our results demonstrate room temperature quantisation of conductance in silicon oxide resistive switches, implying ballistic transport of electrons through a quantum constriction, associated with an individual silicon filament in the SiO_x_ bulk. Because filaments are formed by a valence change process they are less conductive than if they were metallic, and therefore the resulting large Δμ between the electron reservoirs ensures that conductance steps are half-integer multiples of G_0_. This model enables us to resolve a puzzle in the literature over the origin of half-integer quantisation. It predicts which resistive switching systems will exhibit integer or half-integer quantisation, the former being principally metallic ECM devices, the latter predominantly VCM with correspondingly higher resistivity. Furthermore, unlike similar reports of quantisation in metal oxide materials, which are purely linear, our conductance results can be deconvolved into parallel linear and non-linear terms. We associate these with conduction via conductive silicon nanofilaments in parallel with current flow through semiconducting tissue, perhaps surrounding the conductive nanofilament. Our measurements can thus provide useful information on the microscopic structure of the switching layer.

## Methods

### Device fabrication

SiO_x_ layers were deposited at 500°C by magnetron co-sputtering onto p-type, B-doped Si(100) wafers. Two confocal cathodes were used: SiO_2_ and Si, under an argon plasma. The excess silicon content of the films was 11 at.% (measured by XPS: Perkin-Elmer PHI-5500). Samples were annealed at 900°C post-deposition under a nitrogen flux for one hour. 185 nm of n-type silicon (phosphorous doped, resistivity 10 mΩcm) was deposited on top of the SiO_x_ layer by low-pressure chemical vapour deposition (LPCVD). After growth, samples were annealed at 950°C for 30 min in nitrogen to activate the dopants and achieve a final electrode resistivity of 1 mΩcm. Standard photolithographic techniques were used to define top contacts; for the results reported in this study, top electrodes were all 125 μm × 125 μm. The polycrystalline silicon layer was etched by a 20 second immersion in a HNO_3_:H_2_O:HF 50:20:1 mixture. Chrome–gold Ohmic contacts were provided on the back sides of all wafers by evaporation (10 nm Cr, followed by 100 nm Au).

### Electrical measurements

Current/voltage (I/V) data were obtained using a Keithley 4200S Semiconductor Characterisation System and a Signatone probe station. Measurements were two-terminal measurements, with the Ohmic contact grounded and a potential applied to the top contact. Unless otherwise stated, measurements were conducted in an open laboratory in ambient conditions. No current compliance was set for the measurements of quantum conductance; consequently, care was taken to ensure that the measurement regime was well away from the hard breakdown limit of the devices. Low temperature measurements were conducted by immersing samples into liquid nitrogen. Conductance was defined as I/V, and the histogram shown in figure 2(b) was constructed from a measurement of ~1,000 abrupt steps in I/V. Seventy individual devices were measured, for each of which a number of I/V curves was recorded. Some curves yielded more steps than others, but data from all curves were aggregated in order to generate the histogram shown in figure 2. Example curves from nine different devices are shown in the supplementary information to give an indication of the device-to-device variation in conductance jumps.

## Author Contributions

A.M. conceived the study, performed the measurements and wrote the initial draft of the paper; A.V. performed many of the quantised conductance measurements and assisted with data analysis. S.C. grew the active layers and assisted with interpretation; S.H. assisted with electrical measurements and data interpretation; H.H. assisted with the low temperature measurements. C.L. and R.R. oversaw film growth and assisted with data interpretation; M.P. assisted with modelling; A.K. oversaw the project and revised the manuscript.

## Supplementary Material

Supplementary Information Supplementary Information

## Figures and Tables

**Table 1:** Literature reports of quantised conductance in resistive switching systems, showing the division into those systems that show integer and half-integer G_o_ quantisation. V_O_ refers to oxygen vacancies

System	Switching	Filament	Quantisation level
metal/a-Si:H/metal^21^	-	?	½G_0_
V/V_2_O_5_/V^22^	-	?	½G_0_
Nb/ZnO_x_/Pt^10^	ECM	Nb or V_O_	G_0_ or ½G_0_
ITO/ZnO_x_/ITO^10^	VCM	V_O_	½G_0_
W/CeOx/SiO_2_/NiSi_2_^13^	VCM	V_O_	½ G_0_
Ag/Ta_2_O_5_/Pt^23^	ECM	Ag	G_0_
Ag_2_S or Cu_2_S (vacuum gap)^24^	ECM	Ag	G_0_
Ag/Ag_2_S/Pt (STM tip)^25^	ECM	Ag	G_0_
Pt/AgI/Ag^26^	ECM	Ag	G_0_
Ag/GeS_2_/W^27^	ECM	Ag	G_0_
Pt/HfO_2_/Pt^28^	VCM	V_O_ or metallic	G_0_
