# Supplementary material for: Quantum Conductance in Silicon Oxide Resistive Memory Devices
Source: Sci Rep. 2013 Sep 19;3:2708. doi: 10.1038/srep02708 (PMC3776960; doi:10.1038/srep02708)
Supplement: Supplementary Information [file srep02708-s1.doc]

**Supplementary Information**

**Quantum Conductance in Silicon Oxide Resistive Memory Devices**

A. Mehonic^1*^, A. Vrajitoarea^1^, S. Cueff^2,3^, S. Hudziak^1^, H. Howe^1^, C. Labbé^2^, R.Rizk^2^, M. Pepper^1^, A.J. Kenyon^1*^

^1^ Department of Electronic & Electrical Engineering, UCL, Torrington Place, London WC1E 7JE, UK.

^2^ Centre de Recherche sur les Ions, les Matériaux et la Photonique (CIMAP), UMR 6252 CNRS/CEA/Ensicaen/UCBN , 6 Boulevard Maréchal Juin, 14050 Caen Cedex 4, France.

^3^ Brown University, School of Engineering, Providence, Rhode Island 02912, USA

Correspondence and requests for materials should be addressed to AM ([a.mehonic@ee.ucl.ac.uk](mailto:a.mehonic@ee.ucl.ac.uk)) or AK ([t.kenyon@ucl.ac.uk](mailto:t.kenyon@ucl.ac.uk))

1. **Forming**

Prior to switching, devices must undergo an electroforming step, as is common for many resistive switching systems. This serves as an initial nucleation of filaments within virgin material. By applying an appropriate voltage across a fresh device, filament growth is initiated and the device switches from an essentially insulating state (IS) to a low resistance state (LRS). A subsequent reset of the device changes it to a high resistance state (HRS) with a resistance lower than that of the initial virgin material. Further SET – RESET processes cycle the material between the HRS and LRS. Note that the conductivity of the filament formed after switching is strongly dependent on the compliance limit set during the electroforming step[[1]](#endnote-2).

Figure 1 shows an initial electroforming event for one of our devices, which occurs at around -5.5V.


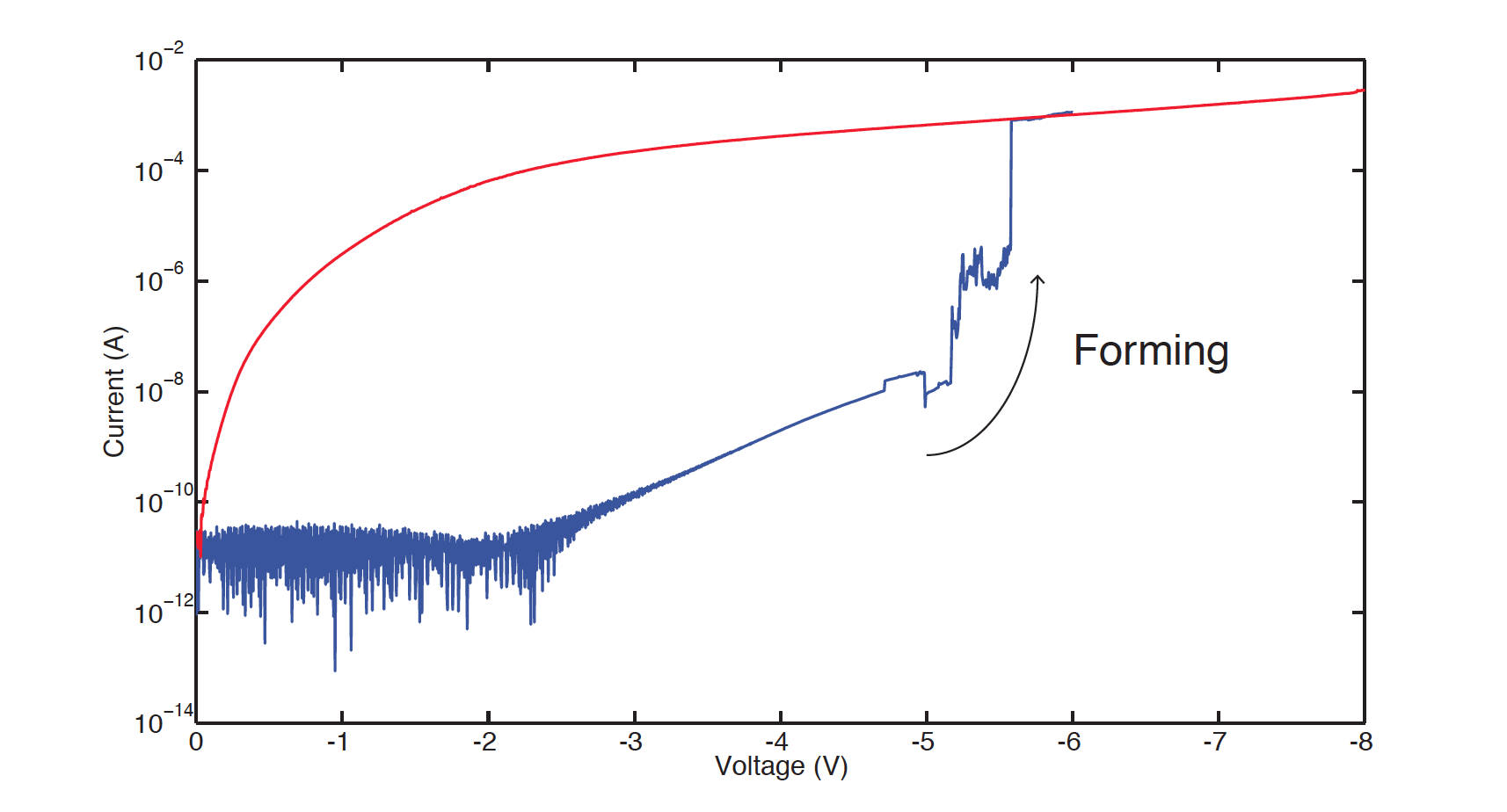


**Figure 1. Electroforming of device conduction, followed by resistive switching.** The device is initially in a high resistive state (HRS – blue line). Increasing the voltage produces a transition to a low resistance state (LRS) at around 5.5V, at which point a filament has formed. Subsequent reset operations do not return the device to its initial state, suggesting that the SET-RESET transition does not involve the complete destruction of the conductive filament.

1. **Parallel conduction paths**

Figure 2 shows two conductance-voltage curves (G_1_ and G_2_) measured sequentially on the same device. Curve G_1_ exhibits an abrupt change in conductance of 5/2G_0_ at a threshold voltage of ~6.6V. The subsequent voltage sweep (G_2_) shows the device has moved to a lower resistance state. Note that the both curves are highly nonlinear; we may therefore model the device conductivity as parallel linear and nonlinear components, with the linear component due to a highly conductive filament core, and the nonlinear component due to conduction through semiconducting tissue surrounding the core. A subtraction of the two curves (G_2_-G_1_) yields a change only in the linear component, as switching will affect only the conductive filament core, and should have little effect on the surrounding semiconducting tissue - as is confirmed by our results reported in the main body of this article. In case of Valence Change Memory (VCM) or Thermochemical Memory (TCM) systems such as that described here, switching occurs at a highly localised region of the conductive filament – it is not a case of the whole filament being disrupted[[2]](#endnote-3). Instead, only a fraction of the filament at a weak point is altered (red section in Figure 2(b)). Such a weak point is likely to be at one of the electrodes. We note that an increasing nonlinear component is also observed for metallic nanocontact break junction devices in which the conductance quantization is analyzed at room temperature[[3]](#endnote-4). In this case it was reported that the nonlinear component is almost independent of the nanocontact conductance and more pronounced at the higher voltages, much like in our system.

For an ideal system, the result of the G_2_-G_1_ subtraction would be a step of magnitude 5/2G_0_. However, at low voltages there is considerable nonlinearity in the G/V curve due to silicon band bending, as has been previously described in the work of Suñé et al[[4]](#endnote-5). Nevertheless, for voltages between 3.5V and 6.5V there is good agreement with the expected result (Figure 1(a) red line), confirming that switching affects primarily the largely Ohmic highly conductive filament core.

An equivalent circuit of the overall cell resistance is shown schematically in Figure 2(c). It is a parallel of the nonlinear component (R_nonlin_) that arises from background conduction of semiconducting tissue surrounding the conductive filament core and switching resistance R_switch_.


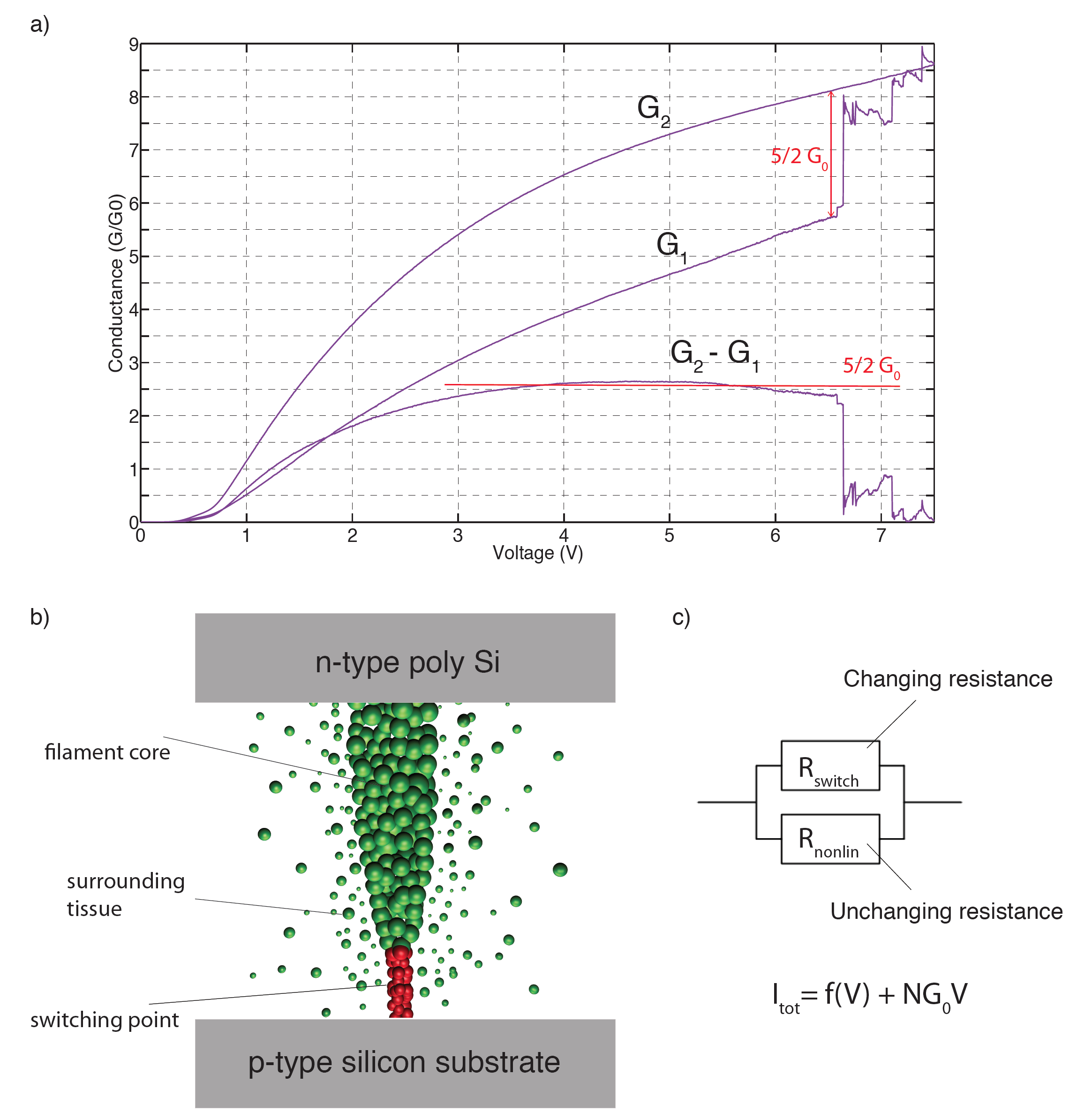


**Figure 2. (a)** Conductance-voltage curves (G_1_ and G_2_) taken sequentially for a single device, showing an abrupt transition of 5/2G_o_ at ~6.6V. Subtracting these curves produces a step of 5/2Go, demonstrating the deconvolution of the linear switching component from the overall nonlinear conductance. **(b)** Schematic of filament, showing the quantum constriction close to one of the electrodes. R_switch_ is the resistance of the quantum constriction, and R_nonlin_ represents the background nonlinear resistance of semiconducting tissue surrounding the highly conductive filament core. **(c)** Equivalent electrical circuit of the schematic shown in 1(b).

1. **Low temperature measurements**

To further examine conduction in the ON state we performed low temperature measurements down to 77K. If the overall conduction consists of two components - linear (Ohmic) and non-linear (semiconducting) - the semiconducting component will be significantly decreased at 77K as free carriers are frozen out. Figure 2 shows current-voltage curves taken at 77K and at room temperature. In contrast to conduction at the room temperature, that at 77K is largely linear for voltages greater than -2.25V, and the overall conduction given by the gradient corresponds closely to one quantum of conductance (G_0_ = 7.75×10^-5^S); the slightly higher measured value (8.1×10^-5^S) can be explained by residual free carriers at 77K.

Note also that when the sample was cooled from room temperature to 77K the overall current measured at a bias of -5V, decreased from 1.87mA to 0.23mA. The overall decrease in the conductivity (by a factor of approximately 8) is too small to be related to strongly temperature-dependent conduction mechanisms such as Poole-Frenkel emission or Thermionic emission where current density *J* depends on temperature as , or to be related to conduction through an intrinsic semiconductor in which the concentration of carriers depends on temperature as , where *E_g_* is the silicon band gap.

However, if the filament is a highly conductive Si-II or Si-XII phase, as suggested by Yao et al[[5]](#endnote-6), or a dangling bond related mini band, as suggested by Wang et al[[6]](#endnote-7), such a result is to be expected as the filament core would behave metallically and have significant conductivity even at low temperatures.

The unipolar switching mechanism in our devices is thermochemical in that Joule heating is required to reset devices, and local temperature plays a role in increasing the mobility of oxygen ions and/or oxygen vacancies during the set process. Consequently, we have not observed set/reset processes or current jumps at low temperatures (eg 77K).

**Figure 3. Temperature dependence of current-voltage characteristics.** Current-voltage curves for a single device measured at room temperature (red line) and at 77K (green line) show a reduction in the nonlinear conduction component at low temperature, consistent with the reduction in free carriers in semiconducting tissue. **Inset:** the gradient of the 77K curve yields a conductance of 8.1×10^-5^S, close to G_o_ (7.75×10^-5^S). The small difference can be due to residual conduction through semiconducting tissue.

1. **Reproducibility of G-V curves**

As stated in the main text, data for the histogram of conductances came from measurements of conductance-voltage curves from seventy individual devices. Several voltage sweeps were recorded for each, from which conductance jump data was extracted. The number of jumps varied from sweep to sweep and between devices, but the final histogram is the result of the aggregation of 1,000 individual conductance steps. Below we reproduce G-V curves for nine representative devices. Despite the variation between individual curves, statistical analysis of conductance data from these, and 61 further devices, yields the histogram shown in figure 1 in the main text.

**Figure 4. Representative conductance-voltage curves from nine different devices.** Data for the conductance histogram in the main text came from such measurements performed on 70 samples. Despite the variation in individual curves, clear conductance steps are apparent, which, when statistically analysed, exhibit quantised steps.

**References:**

1. Ielmini, D. Modeling the universal set/reset characteristics of bipolar RRAM by field-and temperature-driven filament growth. *IEEE T.* *Electron. Dev*, **58**, 4309 (2011). [↑](#endnote-ref-2)
2. Waser, R., Bruchhaus, R. & Menzel, S., in Waser, R. (Ed.) *Nanoelectronics and information technology*, 3^rd^ edition, Wiley-VCH (2012). [↑](#endnote-ref-3)
3. 1. Costa-Krämer, J. L., García, N., García-Mochales, P., Serena, P. A., Marqués, M. I., & Correia, A. Conductance quantization in nanowires formed between micro and macroscopic metallic electrodes, *Phys. Rev*. B, **55**, 5416 (1997).

   [↑](#endnote-ref-4)
4. Suñé, J., Miranda, E., Nafría, M. & Aymerich, X. Modeling the breakdown spots in silicon dioxide films as point contacts, *Appl. Phys. Lett*. , **75**, 959 (1999). [↑](#endnote-ref-5)
5. Yao, J., Zhong, L., Natelson, D. & Tour, J.M. In situ imaging of the conducting filament in a silicon oxide resistive switch. *Sci. Rep.* **2**, 242 (2012). [↑](#endnote-ref-6)
6. Wang, Y., Qian, X., Chen, K., Fang, Z. & Li, W. Resistive switching mechanism in silicon highly rich SiOx (x<0.75) films based on silicon dangling bonds percolation model. *Appl. Phys. Lett*. **102**, 042103 (2013). [↑](#endnote-ref-7)
